# Supplementary material for: Highly efficient transgenesis with miniMos in Caenorhabditis briggsae
Source: G3 (Bethesda). 2022 Sep 28;12(12):jkac254. doi: 10.1093/g3journal/jkac254 (PMC9713419; doi:10.1093/g3journal/jkac254)
Supplement: jkac254_Supplemental_Figures [file jkac254_supplemental_figures.pdf]

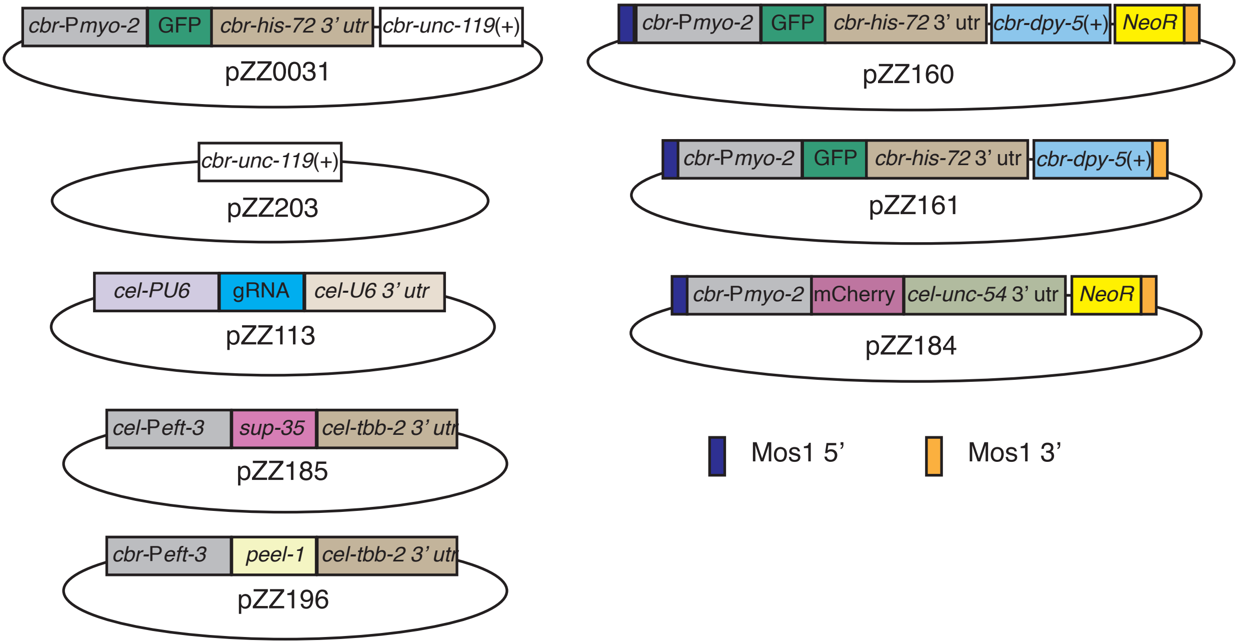

cbr-Pmyo-2
GFP
cbr-his-72 3' utr
cbr-unc-119(+)
pZZ0031
cbr-unc-119(+)
pZZ203
cel-PU6
gRNA
cel-U6 3' utr
pZZ113
cel-Peft-3
sup-35
cel-tbb-2 3' utr
pZZ185
cbr-Peft-3
peel-1
cel-tbb-2 3' utr
pZZ196
cbr-Pmyo-2
GFP
cbr-his-72 3' utr
cbr-dpy-5(+)
NeoR
pZZ160
cbr-Pmyo-2
GFP
cbr-his-72 3' utr
cbr-dpy-5(+)
pZZ161
cbr-Pmyo-2
mCherry
cel-unc-54 3' utr
NeoR
pZZ184
Mos1 5'
Mos1 3'

A

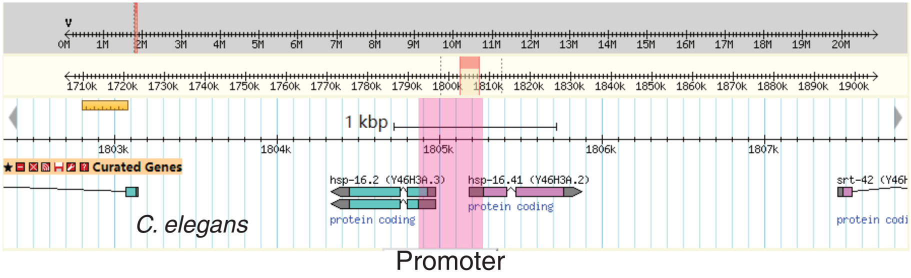

cel-hsp-16.41 promoter region (two back-to-back hsp genes)

Gene Pair	HSP-16.2 Like	HSP-16.41-Like	Direction	Promoter Region
cbr-HSP pair1	CBG19184	CBG19185	Forward	V:13058628..13058983
cbr-HSP pair2	CBG19186	CBG19187	Forward	V:13062275..13062639
cbr-HSP pair3	CBG04591	CBG04592	Forward	V:14582654..14583005
cbr-HSP pair4	CBG04606	CBG04605	Reverse	V:14633959..14634291
cbr-HSP pair5	CBG04608	CBG04607	Reverse	V:14636164..14636508

C. briggsae HSP homology pairs

HSP promoter with the highest similarity to the *cel-hsp-16.41* promoter (highlighted in brown)

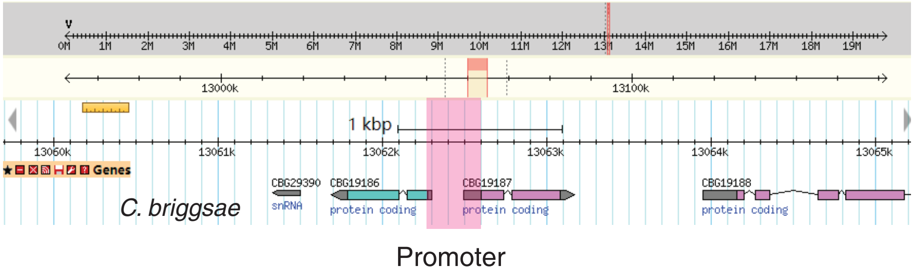

B

> V:13062275..13062639 (*C. briggsae* **genomic region (cb4))**
TTTGGGAGTTGAATCTGAGAAACTGAATGTAGGTTAAGCAGAGGTTCCATGTCTCAGTATTTATAT
GTTTTGCAAGGCCTTTCTAGAATCTTCCAGCTGTTTCTTTTGGCCTCTTTCATCATTCGAACGGA
CAACAACAAAGGAGAAAGCGCGCGGTTGGAAGAGACGCAGACACTTTCTTGTCTGCGTCTCTC
ATTGTGTTGAGGGAGCGTGTACCTTCAGGAATGTTCTAGAAAGTCATCAGGGTTTGATATATATAG
ACTGGTTCAGATGAGAGCAACATACTTTTTTGAAAAGTTCCAAACCTTCTTCCACTTCAAAAATG
CTTCTTCTCCATTCCCCATTCTCTCGTGATAATTGCAAT
